# Conradi's Medium

**Published:** 1909-10-02

**Authors:** 


### CONRADI'S MEDIUM.

If it were easy to grow typhoid bacilli in pure culture from materials containing them in association with myriads of other bacteria, including bacillus coli communis, it would be a great advantage to the profession. Typhoid bacilli could then be detected at once by direct culture from fæces, urine, drinking water, oysters, and so forth. Many attempts at discovering what might be called a " differential culture medium " have been made; and although none is yet perfect, that of Conradi is of considerable use. This observer submitted over 400 aniline compounds to exhaustive tests in order to discover a combination which might be least hurtful to the typhoid bacillus and most inhibitory to other organisms. The outcome of his researches so far is that a mixture containing 1 in 15,000 picric acid and 1 in 150,000 brilliant green (tetra-ethyl diamido-triphenyl carbinol-sulphate) gives the best result. The precise formulary for Conradi's medium is as follows:—Mix together Agar 30 grammes, Liebig's extract 20 grammes, peptone 10 grammes, water 1000 cubic centimetres; add normal sodium hydrate solution or phosphoric acid solution as the case may be until the reaction becomes + 30, Eyre's scale, phenolphthalein indicator; sterilise, and add 1 cubic centimetre of a 1 in 1000 aqueous solution of brilliant green crystals, extra pure Höchst, and 1 cubic centimetre of a 1 in 100 solution of picric acid for each 150 cubic centimetres of the agar mixture.

The typhoid colonies appear as smooth-edged, round, almost flat growths, and they may be immediately picked out and forthwith identified by testing them against an agglutinating typhoid serum. The advantage of this is that the organisms can be diagnosed in a single day.

